# Characterization of Biological Properties of Dental Pulp Stem Cells Grown on an Electrospun Poly(l-lactide-*co*-caprolactone) Scaffold

**DOI:** 10.3390/ma15051900

**Published:** 2022-03-03

**Authors:** Julia K. Bar, Tomasz Kowalczyk, Piotr G. Grelewski, Sandra Stamnitz, Maria Paprocka, Joanna Lis, Anna Lis-Nawara, Seongpil An, Aleksandra Klimczak

**Affiliations:** 1 Department of Immunopathology and Molecular Biology, Medical University, Bujwida 44, 50-345 Wroclaw, Poland; piotr.grelewski@umed.wroc.pl (P.G.G.); anna.lis-nawara@umed.wroc.pl (A.L.-N.); 2 Laboratory of Polymers and Biomaterials, Institute of Fundamental Technological Research (IPPT PAN), Polish Academy of Sciences, Adolfa Pawińskiego 5B St., 02-106 Warsaw, Poland; tkowalcz@ippt.gov.pl; 3 Laboratory of Biology of Stem and Neoplastic Cells, Hirszfeld Institute of Immunology and Experimental Therapy Polish Academy of Sciences, R. Weigla 12, 53-114 Wroclaw, Poland; sandra.gromolak@hirszfeld.pl (S.S.); maria.paprocka@hirszfeld.pl (M.P.); aleksandra.klimczak@hirszfeld.pl (A.K.); 4 Department of Maxillofacial Orthopaedics and Orthodontics, Medical University, Krakowska 26, 50-425 Wroclaw, Poland; joanna.lis@umed.wroc.pl; 5 Department of Nano Engineering & SKKU Advanced Institute of Nanotechnology (SAINT), Sungkyunkwan University (SKKU), Suwon 16419, Korea; esan@skku.edu

**Keywords:** hDPSCs, poly(l-lactide-*co*-caprolactone), electrospun scaffold, biocompatibility, adhesion, proliferation, osteogenic differentiation, tissue engineering

## Abstract

Poly(l-lactide-*co*-caprolactone) (PLCL) electrospun scaffolds with seeded stem cells have drawn great interest in tissue engineering. This study investigated the biological behavior of human dental pulp stem cells (hDPSCs) grown on a hydrolytically-modified PLCL nanofiber scaffold. The hDPSCs were seeded on PLCL, and their biological features such as viability, proliferation, adhesion, population doubling time, the immunophenotype of hDPSCs and osteogenic differentiation capacity were evaluated on scaffolds. The results showed that the PLCL scaffold significantly supported hDPSC viability/proliferation. The hDPSCs adhesion rate and spreading onto PLCL increased with time of culture. hDPSCs were able to migrate inside the PLCL electrospun scaffold after 7 days of seeding. No differences in morphology and immunophenotype of hDPSCs grown on PLCL and in flasks were observed. The mRNA levels of bone-related genes and their proteins were significantly higher in hDPSCs after osteogenic differentiation on PLCL compared with undifferentiated hDPSCs on PLCL. These results showed that the mechanical properties of a modified PLCL mat provide an appropriate environment that supports hDPSCs attachment, proliferation, migration and their osteogenic differentiation on the PLCL scaffold. The good PLCL biocompatibility with dental pulp stem cells indicates that this mat may be applied in designing a bioactive hDPSCs/PLCL construct for bone tissue engineering.

## 1. Introduction

Physiologically, adult bones undergo continuous remodeling through a specific osteoblast/osteoclast interaction. This homeostasis is likely to be disturbed in degenerative disease, after trauma or after surgical procedures causing bone loss [1,2]. Since stem/progenitor cells residing in the periosteum and endosteum have a limited potential to repair bone damage, stimulating the endogenous regenerative process with an innovative approach such as tissue engineering seems to be a promising strategy [2,3,4,5,6,7]. Bone tissue engineering strategy combines three essential components such as scaffolds, mesenchymal stem cells (MSCs) and growth factors, and is based on the culture of stem or progenitor cells on scaffolds in order to generate new bone by osteoinductive cues [3,6,7,8,9]. Generally, the mesenchymal stem cells isolated from bone marrow (BM-MSCs) are the main source of cells for bone tissue engineering, but there still exist concerns regarding their osteogenic efficiency [1]. Moreover, the isolation of autologous stem cells from bone marrow is an invasive and painful procedure, so they have limited clinical application for tissue engineering [8,10,11].

Human dental pulp stem cells (hDPSCs) have many features resembling BM-MSCs and can be easily isolated using non-invasive techniques required for MSCs collected from bone marrow [2,12,13,14]. hDPSCs possess MSCs characteristics defined according to the International Society for Cellular Therapy (ISCT) criteria [15]. The osteogenic potential of hDPSCs was intensively investigated in pilot clinical studies where autologous hDPSCs were successfully used in patients with a periodontal bone defect or a post-third molar extraction defect and for dental pulp regeneration [16,17,18].

Several bi-dimensional (2D) and three-dimensional (3D) natural and synthetic scaffolds and their mixed components blends have been tested for bone tissue engineering, but an ideal scaffold is not yet developed due to the fundamental requirement for tissue-engineered bone grafts, which enables the ability to integrate with the host tissues and promote their repair [8,9,10]. Scaffold design for bone tissue engineering involves many parameters such as physical, mechanical, chemical and biological factors that affect scaffold properties and have an impact on stem cells behavior [8,9,11]. It was found that 2D scaffolds are unable to support in vitro cell growth and organization in a tissue-like structure because of the lack of the extracellular matrix (ECM) providing a three-dimensional (3D) microenvironment for the cells in vivo [10]. Three-dimensional scaffolds provide chemical and physical properties to guide tissue development, and the internal architecture of the scaffold should serve as a substrate for stem cell–cell communication and interaction with a platform to form a functional bioengineered construct [7,8,11,19]. Biomaterials used to design the 3D scaffold should be biocompatible, allow for oxygen and nutrient transport, cell attachment and growth without an immune response, and the degradation products should not be toxic [11,20,21]. Moreover, a biomaterial needs to have good tensile and compressive strength, be osteoinductive and promote proliferation and ECM generation [10,11]. Furthermore, a scaffold for bone tissue engineering should induce angiogenesis because new blood vessel formation is necessary for nutrients and oxygen transport [11,22]. Usually, scaffolds developed for bone tissue engineering try to mimic the natural matrix, and the most frequently used are natural and synthetics polymers, ceramics and composites [8,9,11,23]. Natural polymers consist of proteins, and among the natural polymers, collagen is most frequently used as a matrix in bone regeneration [23,24]. The scaffold consists of natural materials that show low antigenic response, high tensile strength and high flexibility, but have the disadvantages of mechanical weakness and instability, the risk of transmitting pathogens and they may induce immune response if not properly purified [9,20,23,25]. Synthetic biomaterials provide an alternative to natural materials for bone tissues engineering and include polymer-based polylactic acid (PLA), poly-ε-caprolactone (PCL), poly (ethylene glycol) (PEG), poly (lactic-*co*-glycolic acid) (PLGA) and ceramics-based biomaterials such as hydroxyapatite, bioactive glass and can be produced in controlled conditions, optimizing the chemical and physical properties of a scaffold [11,23]. These materials offer many advantages over their natural counterparts, including reproducibility and the ability to control the mechanical properties, degradation rate and shape independently [25]. However, many synthetic biomaterials lack cell adhesion properties and must be chemically modified to allow for stem cell adhesion and growth [8,9,23]. Ceramics are characterized by their high toughness and biocompatibility, although they are vulnerable to stress tensile and can be damaged due to excessive mechanical stress [25]. Hybrid composites are also used as promising biomaterials for bone regeneration. The combination of composites used for scaffold fabrication allow each biomaterial to bring features lacking in other ones, and the advantageous properties can be combined in order to obtain more specific chemical, physical and mechanical properties [11,23,26]. A scaffold design of composites presenting different features may show better mechanical stability, flexibility and structural integrity [27]. Other important features of a scaffold which have impact on the metabolic dispersion and the cell migration rate within the scaffold is its porosity [9,10].

The density and size of pores should allow for cell spreading and adherence to the scaffolds, as well as nutrient and oxygen diffusion [28,29,30]. Scaffolds with higher porosity often show higher permeability and cell infiltration but can lead to poor mechanical resistance and can make a scaffold too weak [28,31]. Whereas, excessively large pores can be prejudicial to the mechanical properties of the structure and discourage the ECM synthesis between the fibers [28,31]. However, smaller pores can promote adequate cell adhesion and short-term proliferation, but they are associated with the formation of non-mineralized osteoid or fibrous tissue [28,31,32]. Different fabrication techniques are used to obtain a scaffold with proper architecture and mechanical properties for tissue engineering [10,33]. The electrospinning technique is considered to have unique advantages over some other scaffold fabrication techniques because it can generate fibrous scaffolds ranging in size from nanometers to micrometers [25,33]. The high porosity, porous structure and large surface area of electrospun nanofibrous scaffold could potentially mimic the natural ECM of biological tissues [8,33].

To date, for regenerative purposes, hDPSCs used for tissue and bone reconstruction are seeded on biocompatible and bioactive natural and synthetic biomaterials, such as collagen, chitosan, silk, alginate, hyaluronic acid, poly(lactide) and their derivatives [5,34,35,36]. Studies showed that although resorbable solid membranes have many beneficial properties thanks to being hemostatic and chemotactic, they are too unstable, do not guarantee porosity to ensure complete bone repair and do not sufficiently stimulate of hDPSCs osteogenic differentiation [3,7,17,19,37,38]. 

To overcome these problems, recently, bioengineering has focused on looking for ideal materials as scaffold components and adequate techniques for its fabrication [5,39,40,41]. Subject literature includes reports on electrospinning techniques used to fabricate a nanofibrous mat with appropriate pore size and internal/external scaffold geometry suitable for bone regeneration [5,38,39]. There are numerous studies on the use of nanofibrous mats produced from bioresorbable synthetic polymers (e.g., poly(-caprolactone) and poly(-l-lactide), copolymers (e.g., poly(-l-lactide-*co*-glycolide) and natural polymers (e.g., gelatin) combined with stem cells [5,38,39,40,42]. It was determined that nanofibrous mats enable easy access to oxygen and nutrients and drain metabolites [39]. Furthermore, although the hydrophobic surface of fibers is not colonized by the cells, modifying the surface makes the fibers feasible for cell attachment and proliferation [5,39,43]. There are only a few reports analyzing the biological potential of a pure poly(-l-lactide-*co*-caprolactone) (PLCL) mat used for the regeneration of the skin and the abdomen and uterus reconstruction in an animal model [41,44]. However, a pure PLCL scaffold is hydrophobic, and some modifications are needed to improve its physicomechanical properties [42,44,45]. Recently, several reports found that the modification of electrospun poly(l-lactide) or poly(ε-caprolactone) nanofiber scaffolds improves hDPSCs osteogenic differentiation [5,39,46]. Although electrospun nanofibers scaffolds showed high potential in bone tissue engineering, different problems must be solved. Scaffolds fabricated with natural polymers mainly suffer from low mechanical strength and structural integrity, whereas synthetic polymers possess good mechanical properties, but they are not suitable for cell attachment due to their low biological compatibility [9]. Therefore, polymeric nanofiber scaffolds still need further optimization of their composition and structure for in vivo applications [33]. Other issues being mainly related to viable microenvironment to boost the development of dental stem cells towards new tissue formation is the lack of functional vascular supply upon implantation and even the sealing of the pores of the scaffold [47]. The scaffold should be porous to distribute an optimal number of stem cells to develop tissue [48,49]. In light of the above limitations connected with the selection of scaffold components and their fabrication as well as MSCs source, we analyze the impact of a hydrolytically modified hybrid electrospun nonwoven PLCL mat on the biological behavior of hDPSCs growing on the nanofiber scaffold. Based on the possibility that the latter has the appropriate porosity and mechanical parameters to generate an environment supporting hDPSC attachment, proliferation, migration and osteogenic differentiation, verifying this null hypothesis became the aim of our study.

## 2. Materials and Methods

### 2.1. Materials

The material used in this study was a PLCL nanofiber mat (Purasorb 7015, Purac-Corbion, Amsterdam, The Netherlands) composed of 70% L-lactide and 30% caprolactone units with an inherent viscosity of 1.5 dL/g, which is GMP certified and widely used for the production of medical devices.

#### 2.1.1. Electrospinning and Hydrolytic Modification

Electrospinning was carried out in a Fluidnatek LE-50 chamber equipped with a humidity and temperature control module (Bioinicia, Valencia, Spain). A 9% PLCL solution dissolved in a mixture of chloroform and dimethylformamide (1:16 *w*/*w*) was prepared by magnetically stirring for half a day to achieve a homogenous electrospinnable solution. The operating conditions for electrospinning the PLCL solution were as follows: flow rate 500 μL/h, nozzle-to-collector distance 14.5 cm, applied voltage 15 kV, electrospinning time 3 h, temperature 25 °C, relative humidity 38% and rotating speed of the drum collector 500 min^−1^. Note that the electrospun PLCL nanofiber mat was left for half a day attached to the drum collector at a temperature of 37 °C. The PLCL nanofiber mat was hydrolytically modified by dipping it in a 10% NaHCO_3_ aqueous solution for four days at 37 °C.

#### 2.1.2. Characterization

The molecular weight and dispersity of the polymer were measured using Gel Permeation Chromatography (GPC, Nexera LC-40, Shimadzu, Kyoto, Japan). The morphologies of the PLCL nanofiber mat were characterized using a scanning electron microscope (SEM, JSM 6390 LV, Jeol, Tokyo, Japan) with 10 kV accelerating beam energy. The fiber size distribution of the electrospun PLCL nanofibers was determined by measuring 100 fibers in top-view SEM images. The thickness of the electrospun PLCL nanofiber mat was also measured by analyzing cross-sectional SEM images. The SEM specimens were prepared by cutting the nanofiber mat with sharp scissors at room temperature (RT). Note that, prior to the SEM analysis, the specimens were sputtered with gold (Mini Sputter Coater, SC 7620, Polaron, London, UK). The contact angle of the specimens was measured using a goniometer (OCA 15, DataPhysics Instruments, Filderstadt, Germany). The porosity of the specimens was also determined by comparing the total mass of the nanofiber mat with a neat polymer specimen of the same size.

### 2.2. Stem Cell Study-Related Methods

#### 2.2.1. Patients

Human dental pulp was extracted from the third molars of four healthy individuals (aged 15–32 years) undergoing routine tooth extraction. All participants of the study provided informed signed consent following a detailed explanation of the research procedure. All procedures regarding tissue collection and in vitro analysis of dental pulp stem cells were approved by the Ethics Committee of the Medical University in Wroclaw, Poland (No. KB-642/2017). 

#### 2.2.2. hDPSC Isolation

Before the tooth extraction, oral cleaning and disinfection using diluted povidone-iodine was performed. After the extraction, the teeth were cut at the cementum–enamel junction using a sterilized diamond bur, and dental pulp tissue was gently removed from the tooth and immersed for 1 h at 37 °C in a digestive solution containing 3 mg/mL of collagenase type I from *Clostridium histolyticum* (Sigma-Aldrich, St. Louis, MO, USA) and 4 mg/mL of Dispase II from *Bacillus polymyxa* (Gibco, Life Technologies, New York, NY, USA). After digestion, the samples were filtrated through a 70 µm pore size cell strainer and centrifuged at 400× g for 10 min (Falcon, Corning, New York, NY, USA) [13]. 

#### 2.2.3. hDPSC Culture

hDPSCs from four donors were cultured in culture flasks/dishes at 37 °C and 5% CO_2_ in the α-minimal essential medium (α-MEM; Gibco, Karlsruhe, Germany) supplemented with 20% fetal calf serum (FBS) (Gibco, Karlsruhe, Germany) and antibiotics (100 IU/mL penicillin and 100 µg/mL streptomycin; (Sigma-Aldrich, St. Louis, MO, USA). The medium was changed every three days. When the dental pulp stem cells reached 90% confluence, they were passaged using a 0.25% trypsin-EDTA solution (Sigma-Aldrich, St. Louis, MO, USA). At the third passage, the cells were evaluated using flow cytometry to detect mesenchymal stem cell markers [12]. The MSC characteristics of the isolated hDPSCs were confirmed according to the criteria of the International Society for Cellular Therapy [15]. The three main features of hDPSCs were as follows: adherence to plastic, expression of specific CD markers, and trilineage differentiation potential for chondrogenesis, osteogenesis, and adipogenesis [15]. All experiments were performed using hDPSCs at the third passage in two replicates. 

#### 2.2.4. hDPSC Phenotype Identification

##### Flow Cytometry Analysis

The phenotype of primary hDPSCs (*n* = 4) at passage 3 (P3) was characterized using flow cytometry. Briefly, 2 × 10^5^ cells for each marker were resuspended in 50 µL of the PBS buffer and incubated with selected human-specific monoclonal antibodies, directly labeled with PE: CD73 (clone AD2), CD90 (clone 5E10), CD105 (clone 266), CD45 (clone HI30), CD31 (clone WM59), CD54 (ICAM-1) (clone HA58), CD106 (VCAM-1) (clone 51-10C9), HLA ABC (clone G46-2.6), HLA DR (clone G46-6) (all from BD Pharmingen, San Jose, CA, USA), CD44 (IgG2a, clone 156-3C11), (Thermo Fisher Scientific, Rockford, MI, USA) and Stro-1 (clone STRO-1) (Invitrogen, Thermo Fisher Scientific, Rockford, MI, USA). All samples were incubated for 30 min in the dark at 4 °C. Mouse isotype-matched IgG_1_ and IgG_2_ were applied as negative controls (BD Pharmingen, San Jose, CA, USA). The cells were analyzed using flow cytometry with a FACS Calibur platform (Becton Dickinson, San Jose, CA, USA). The expression levels of the selected antigens were evaluated using the CellQuest software (Becton Dickinson, San Jose, CA, USA).

#### 2.2.5. Multilineage Differentiation Potential

##### Chondrogenic Differentiation

For chondrogenic differentiation, hDPSCs from four donors were seeded at a density of 8 × 10^3^ cells per well in a 6-well plate and cultured in α-MEM complete medium. When the hDPSCs reached 90% confluence, the cultured medium was removed, and the cell pellet was maintained in hMSC chondrogenesis induction medium, serum-free kit (Provitro, Berlin, Germany). hDPSCs were cultured for 28 days in a chondrogenesis induction basal medium containing dexamethasone, sodium pyruvate, ascorbic-acid-2-phosphate, proline and antibiotics, which was supplemented by the human transforming growth factor-beta 3 (TGF-β-3). Growth factors were added fresh daily in concentration 10 µL/mL induction medium. The culture medium was changed twice a week. The status of the differentiated hDPSCs was confirmed using Alcian Blue (Abcam, Inc, Cambridge, UK) staining. After 28 days of culture, the cells were washed with PBS, fixed in 10% formalin solution (Merck, Saint Louis, MO, USA), washed in PBS again and incubated in the Alcian Blue solution for 30 min. In the next step, the dishes with the differentiated cells were washed once in running tap water and twice in distilled water [50]. The dishes with the stained cells were analyzed using an Olympus IX73 microscope (Olympus, Tokyo, Japan). 

##### Osteogenic Differentiation

For osteogenic differentiation of hDPSCs, the stem cells from four donors were seeded at a density of 8 × 10^3^ cells per well in a 6-well plate in α-MEM complete medium and cultured until they reached approximately 60% confluence. Subsequently, the medium was replaced with a complete hMSC osteogenesis induction medium, FCS-kit (Provitro, Berlin, Germany). According to manufacturing protocol, 50 mL osteogenic induction basal medium was prepared by adding 5 mL FCS, 1ml HEPES, 350 µL penicillin and streptomycin, 0.5 mL L-glutamine. Next, supplemented by osteogenic induction factors, 500 µL dexamethasone, 500 µL β-glycerol-phosphate and 500 µL ascorbic acid-2-phosphate were added. hDPSCs were differentiated for 21 days at 37 °C and 5% CO_2_ atmosphere. The induction medium was changed twice a week. After 21 days of cultivation, the status of the differentiated hDPSCs was confirmed using Alizarin Red S (Sigma-Aldrich, St. Louis, MO, USA) staining to detect mineral deposition. After 21 days of culture, the cells were washed with PBS, fixed in 10% formalin solution, washed in PBS again, and incubated with Alizarin Red S for 30 min in a dark chamber at RT [50]. Finally, the dishes with the differentiated cells were washed in PBS and analyzed using an Olympus IX73 microscope (Olympus, Tokyo, Japan).

##### Adipogenic Differentiation

For adipogenesis, hDPSCs (*n* = 4) were seeded in an α-MEM complete medium at a density of 4 × 10^4^ cells per well in a 24-well plate. The culture medium was changed to the adipogenic differentiation medium using a StemPro^®^ Adipogenesis Differentiation Kit (Gibco, Invitrogen, Grand Island, NY, USA) at a volume of 400 µL per well. The medium was changed every three days. After 28 days of incubation, the cells were washed with PBS, fixed for 20 min at RT in a 10% formalin solution and washed with PBS again. To confirm the adipogenic differentiation, the hDPSCs were washed with 60% isopropanol (POCh, Gliwice, Poland), dried for 1 min, and stained with 200 µL of the Oil Red O solution (Merck, Saint Louis, MO, USA) for 20 min. Next, they were washed with 60% isopropanol twice and once with distilled water [50]. The results were visualized using an optical microscope (Primovert, Zeiss, Jena, Germany).

### 2.3. Preparation of the hDPSCs-PLCL Scaffold Construct

The hDPSCs at the density of 2 × 10^5^ were resuspended in 70 µL of culture medium and carefully dripped onto a precut PLCL 0.5 × 0.5 cm PLCL scaffold and placed in Petri dishes and incubated for 2 h at 37 °C and then analyzed under the inverted microscope to check cells adhesion to the scaffold surface. Next, 2 mL of the α-MEM medium was added and cultured for seven days. The medium was changed twice a week. The hDPSCs from all donors (*n* = 4) were used to prepare the hDPSCs-PLCL construct.

#### 2.3.1. Impact of the PLCL Membrane on hDPSC Viability

The survival rate of hDPSCs growing on the nanofibrous scaffold was analyzed using the 3-(4,5-dimethylthiazol-2-yl)-2,5-dipheyltetrazolium bromide MTT assay. In total, 1 × 10^4^ hDPSCs were seeded onto a precut in size 0.3 × 0.3 cm PLCL scaffold, cultured in 96-well microculture plates and incubated at 37 °C for one, three and seven days. hDPSCs at a density 3 × 10^3^ cells/well in 96-well plate cultured at the same time point without a scaffold served as control. Afterwards, the scaffold was removed, hDPSCs were transferred onto 96-well plates and 200µL of 3-(4,5-dimethylthiazol-2-yl)-2,5-diphenyltetrazolium bromide (Sigma-Aldrich, St. Louis, MO, USA) was added before to incubation at 37 °C for 4 h. After the four h of incubation, formazan crystals were dissolved by adding 100 µL of acidic isopropanol (38% HCl in 99.7% isopropanol) [39]. The absorbance value was measured at 570 nm using a GloMax^®^ Discover multimode microplate reader (Promega, Madison, WI, USA). The experiments were performed in three replicates for each hDPSC case (*n* = 4), and the data were calculated as mean + SD.

#### 2.3.2. Adhesion of hDPSCs to the PLCL Scaffold

hDPSCs were seeded onto the PLCL scaffold at a density of 1 × 10^4^/cm^2^ in six-well plates and cultured in the α-MEM medium at 37 °C with 5% CO_2_ for 4 h and 24 h. hDPSCs at a density 1 × 10^3^ cells/well in 6-well plate cultured at the same time point without a scaffold served as a control. The adherent hDPSCs were enzymatically (trypsin 0.25%-EDTA) removed from the scaffolds and counted using an Automated Cell Counter mark R1 (Olympus, Tokyo, Japan). The hDPSC adhesion rate was expressed as a percentage of the initial number of seeded cells. Additionally, the cytoskeleton hDPSCs grown on PLCL mat before and after osteogenic differentiation were determined by actin visualization using phalloidin staining. Stem cells on the PCLC scaffold were fixed for 15 min at RT in 10% formalin solution (Merck, Saint Louis, MO, USA). Next, the cells on the scaffold were treated with 0.1% Triton™ X-100 for 15 min and washed with PBS three times. hDPSCs were incubated with Alexa Fluor 488-phalloidin (Thermo Fisher Scientific, USA) (dilution 5 µL of the methanol stock solution in 200 µL of PBS) for 40 min. Then, the cells were stained with 4′,6-diamidino-2-phenylindole (DAPI). Finally, the hDPSCs adhesion onto the scaffold was observed using a fluorescence microscope (BX61, Olympus, Tokyo, Japan). The experiments were repeated three times for each hDPSC case (*n* = 4).

#### 2.3.3. Assessment of hDPSCs Spread and Population Doubling Time (PDT) onto the PLCL Scaffold

hDPSCs at a density of 1 × 10^4^/cm^2^ were seeded on the PLCL scaffold in 6-well plates and cultured in the α-MEM medium at 37 °C with 5% CO_2_ for one, three, and seven days. At a selected time-point of observation, the hDPSCs-PLCL constructs were removed from the plates, washed with PBS twice for 5 min, and put onto slides. Next, they were fixed in cold (4 °C) methanol for 10 min and left to dry at RT. The cell samples were stained with DAPI for 10 min (ProLong, Thermo Fisher Scientific, USA). hDPSC distribution on the scaffold was observed using a fluorescence microscope (BX61, Olympus, Tokyo, Japan). The number of hDPSCs on the PLCL surface was calculated using the Olympus CellSence Dimension image processing software (Olympus, Tokyo, Japan) [4]. 

The hDPSCs spreading analyses were repeated three times for each hDPSC case (*n* = 4), and the data were reported as mean ± SD.

Population doubling time analysis was performed for each experimental time point. Blue fluorescent stained cells were counted in 10 randomly selected fields within the scaffold, with a surface of 1 mm^2^ each, using the Olympus CellSence Dimension software and a fluorescence microscope (BX61) (Olympus, Tokyo, Japan). hDPSCs density was expressed as the mean of cells/cm^2^ ± SD. The population doubling time (PDT) was calculated according to the following formula [51]:

$$\mathrm{Population}\;\mathrm{doubling}\;\mathrm{time}=\frac{log_{10}\left(N\right)-log_{10}\left(N0\right)}{log_{10}\left(2\right)}$$

where *N* is the number of cells at the end of the procedure and *N*0 is the number of cells at the beginning of culture. The control group were hDPSCs grown as a monolayer (*n* = 4). The density of hDPSCs was calculated based on the dimension of the scaffold and was 1 × 10^4^ cells/well surface area 9.6 cm^2^ or 0.32 cm^2^. The experiments were repeated three times for each hDPSC case (*n* = 4).

#### 2.3.4. Cell Membrane Staining with the PKH26 Fluorescent Dye

The hDPSCs were stained using a fluorescent dye PKH26 Red kit (Sigma-Aldrich, St. Louis, MO, USA) according to the manufacturer’s protocol. Briefly, 2 × 10^7^ of hDPSCs were washed using the serum-free Minimum Essential Medium α-transformation—α-MEM (IIET PAS, Wroclaw, Poland), centrifuged at 400× *g* for 5 min, and resuspended in 1 mL of Diluent C. PKH26 dye solution, which was prepared by adding 4 μL of the PKH26 Red dye to 1 mL of Diluent C. Next, the PKH26 dye solution was added to the resuspended cells and incubated for 5 min at RT. The staining was stopped with 2 mL of FBS (Biowest, Riverside, MO, USA). After 1 min of incubation, the cell suspension was centrifuged at 400× *g* for 10 min. The cell pellet was washed three times in complete α-MEM. The stained cells were seeded at a density of 5 × 10^5^ cells per scaffold onto a 48-well plate in duplicate. Next, the cells were observed for three days using a fluorescence microscope (BX61, Olympus, Tokyo, Japan). To analyze cell proliferation on the scaffold, fluorescence intensity was quantified using Image Software.

To visualize both the lipophilic membrane and the nuclei of the cells, they were additionally stained with DAPI after three days of incubation at the end of the experiment. Next, the scaffolds with the stained cells were transferred onto glass coverslips, and images were captured using a fluorescent microscope (BX61, Olympus, Tokyo, Japan). The experiments were repeated three times for each hDPSC case (*n* = 4). 

#### 2.3.5. Morphological Features and Immunophenotype of hDPSCs Grown on the PLCL Scaffold 

A histological and immunohistochemical examination of the hDPSC/PLCL construct (*n* = 4) was performed. Each sample was fixed in 10% formalin solution (Merck, Saint Louis, MO, USA) for 24 h, dehydrated in serially degraded ethanol, and embedded in paraffin blocks using Bio-Wraps (Leica, Biosystem, Inc., Wetzlar, Germany) according to the conventional method. The paraffin blocks were cut into 5-µm-thick sections, deparaffinized and stained with hematoxylin and eosin to allow for the morphological analyses of the features of the hDPSC loaded on the membrane and grown for seven days [44]. As a control, hDPSCs were cultured for one week as a monolayer in flasks. Afterwards, the cells were trypsinized, and cytospin preparations were developed and fixed in cold acetone (4 °C) for 10 min, stained with hematoxylin and eosin and examined cytologically using a BX51 microscope (Olympus, Tokyo, Japan). The immunophenotype of hDPSCs was analyzed on hDPSCs/PLCL paraffin block sections, the surface of hDPSCs/PLCL construct fixed in 10% formalin solution (Merck) and cytospin specimens using antibodies and immunohistochemical staining (see Section 2.4 and Section 2.4.1) and compared with hDPSCs immunophenotype grown as monolayer. The cytospin specimens were developed by the cells removed from the PLCL scaffold using a 0.25% trypsin-EDTA solution (Sigma-Aldrich, St. Louis, MO, USA). After cells washing in PBS, the cytospin preparations were developed and fixed in 10% formalin solution (Merck, Saint Louis, MO, USA) for 15 min.

#### 2.3.6. Osteogenic Capabilities of hDPSCs Grown on PLCL Scaffold

For the analysis of osteogenic potential, hDPSCs from four donors were seeded at 4 × 10^3^ cells/cm^2^ on PLCL and maintained in a complete hMSC osteogenesis induction medium, FCS-kit (Provitro, Germany), and cultured at 37 °C with CO_2_ for 21 days according to the manufacturer’s protocol as described above (Section Osteogenic Differentiation).

#### 2.3.7. Total RNA Isolation, Reverse Transcription and Quantitative Polymerase Chain Reaction (qRT-PCR)

After 21 days, differentiated hDPSCs on PLCL and undifferentiated hDPSCs/PLCL specimens (*n* = 4) were rinsed with PBS three times, and total RNA was isolated using RNeasy Plus Mini Kit (Qiagen, Hilden, Germany) according to the manufacturer’s protocol. The RNA concentration and purity were determined using a MaestroNano Spectrophotometer (Maestrogen, Inc., Hsinchu, Taiwan). 

The gene expression of osteogenic markers: *osteocalcin*, *osteopontin*, *bone sialoprotein* and *dentin sialophosphoprotein* was evaluated by qRT-PCR (quantitative reverse transcription polymerase chain reaction). The reverse transcription of 0.1 µg total RNA from each sample was used to prepare cDNA with a Quantitect Reverse Transcription Kit (Qiagen, Hilden, Germany). Real-time PCR was performed using Rotor-Gene SYBR Green (Qiagen, Hilden, Germany) on a ViiA 7 Real-Time PCR System (Applied Biosystems, Foster City, CA, USA). Primers used in the reactions are listed in Table 1. The real-time PCR reactions were conducted in triplicate with the program running for *osteocalcin* and *osteopontin*: initial denaturation at 95 °C for 10 min, next 40 cycles of denaturation at 95 °C for 1 min, annealing at 60 °C for 1 min and extension at 72 °C for 40 s; for *bone sialoprotein* and *dentin sialophosphoprotein*: initial denaturation at 50 °C for 15 min and 94 °C for 4 min, next 40 cycles of denaturation at 94 °C for 20 s, annealing at 60 °C for 20 s and extension at 72 °C for 40 s. The normalization of the PCR products was quantified to the housekeeping gene β-actin. The 2^−ΔΔCt^ method was used to calculate the relative mRNA expression level, where threshold cycles (Ct) from triplicate runs were averaged and calibrated to β-actin Ct. The analysis was repeated three times for each hDPSC case (*n* = 4).

### 2.4. Antibodies

Immunohistochemical staining of the CD105, CD90, CD44 and Stro-1 molecules expression was performed using the following antibodies: anti-CD105 (mouse monoclonal, IgG2a, clone MEM-229; 1:300, Thermo Fisher, Scientific, Rockford, MI, USA), anti-CD90 (mouse monoclonal, IgG1, clone AF-9, 1:200, Thermo Fisher Scientific, Rockford, MI, USA), anti-CD44 (mouse monoclonal, IgG2a, clone 156-3C11, 1:4000, Thermo Fisher Scientific, Rockford, MI, USA), anti-STRO-1 (mouse monoclonal, IgM, STRO-1, 1:50, Abcam, Inc, Cambridge, UK), anti-osteocalcin (mouse monoclonal, IgG1, clone (OCG4), 1:400 (Thermo Fisher Scientific, Rockford, MI, USA), anti-osteopontin (mouse monoclonal, clone 7C5H17, 1:200, Abcam, Inc., Cambridge, UK), anti-bone sialoprotein (rabbit polyclonal, 1:50, Abcam, Inc., Cambridge, UK), anti-dentin sialophosphoprotein (rabbit polyclonal, 1:200, Abcam, Inc., Cambridge, UK). 

#### 2.4.1. Immunohistochemical Staining (IHC)

Immunohistochemical staining for the analyzed stem cells markers was performed on paraffin-embedded hDPSC-scaffold specimens, hDPSCs/PLCL construct surface and cytospin slides of hDPSCs before osteogenic differentiation, whereas bone-related proteins expression was analyzed before and after hDPSCs differentiation on PLCL into osteoblasts for all stem cell donors using the Universal Dako REAL EnVision Detection System, Peroxidase/DAB+, Rabbit/Mouse (Dako, Copenhagen, Denmark) and primary antibodies against CD44, CD90, CD105, Stro-1, OCN, OPN, DSPP and BSP. Five μm sections of the hDPSC-scaffold specimens were deparaffinized and boiled for 2 × 5 min in a citrate buffer (pH = 6.0) at 800 W in a microwave. Next, the hDPSC-scaffold sections were slowly cooled for 30 min. Next, endogenous peroxidase reactivity was blocked with the Dako REAL Peroxidase Blocking Solution (Dako, Copenhagen, Denmark), after which the specimens were incubated with primary antibodies overnight at 4 °C. The cytospin specimens were incubated with primary antibodies for 60 min at RT. After washing, with 0.1 M Tris buffer, pH = 7.4 (TBS), the scaffold specimens and cell cytospine were incubated with Dako REAL EnVision/HRP, Rabbit/Mouse (Dako, Copenhagen, Denmark) for 30 min at RT. The antigen-antibody reaction was visualized using DAB (3,3 diaminobenzidine) (Dako, Copenhagen, Denmark) as a chromogen for four min at RT. The sections were counterstained with hematoxylin and mounted. The incubation buffer (TBS) without primary antibodies was used as a negative control. Positive controls for each antibody were performed according to the manufacturer’s recommendation [44]. 

#### 2.4.2. Immunohistochemical Staining Interpretation

The expression of the analyzed proteins in the hDPSCs located on the membrane and cell cytospin was assessed semiquantitatively taking into account the staining intensity and the number of cells showing immunoreactivity for CD44, CD90, CD105, Stro-1, OCN, OPN, DSPP and BSP. The percentage of immunopositive cells was determined by counting positive cells for CD105, CD90, CD44, Stro-1, OCN, OPN, DSPP and BSP versus the total number of cells visible in randomly selected areas of the hDPSCs/scaffold construct. In the cytospin preparation, the percentage of positive cells for the analyzed proteins was determined by counting 1000 cells in randomly selected areas using an Olympus BX51 microscope (Olympus, Tokyo, Japan). Positive staining required more than 5% of cells showing a reaction. The intensity score was based on the color of the reaction, where 0 = no immunostaining, light yellow color = weak (+), medium brown color = moderate (++) and brown color = strong (+++). 

#### 2.4.3. Immunofluorescence Technique

The immunohistochemical staining for CD44, CD90, CD105 and STRO-1 on the hDPSCs from four donors located on the scaffold (*n* = 4) was confirmed using immunofluorescence staining. The hDPSC-scaffold construct was fixed in cold methanol (4 °C) for 10 min and dried. Next, the specimens were incubated with the primary antibodies overnight at 4 °C and washed twice in PBS for 5 min. The membrane specimens were incubated with the secondary antibody TRITC or FITC (Polyclonal Rabbit Anti-Mouse Immunoglobulins TRITC or Immunoglobulins FITC (Dako, Copenhagen, Denmark) for 30 min at 4 °C in the dark. After incubation, the specimens were washed two times in PBS. Negative controls for fluorescence staining have been developed without primary antibody; only the secondary antibody was used: PLCL specimens with hDPSCs and PLCL scaffold alone. Finally, three drops of DAPI were added, and after 5 min, the samples were assessed using a BX61 fluorescence microscope (Olympus, Tokyo, Japan) [4].

### 2.5. Statistical Analysis

Data are represented as mean ± SD. Statistical difference between mean values of MSCs biomarkers and bone-related proteins expression, hDPSCs viability, adhesion and unmodified and modified parameters of PLCL membrane was determined using a student *t*-test. The differences in hDPSCs on PLCL spreading was analyzed using the ANOVA Friedman test, population doubling time using non-parametric U-Mann–Whitney test. Statistical analysis was performed using Stastistica TIBCO Software Inc., version 13 (Palo Alto, CA, USA). To compare data for bone-related genes expression, the one-way analysis of variance (one-way ANOVA) with Dunnet’s test for multiple comparison procedures was used. Statistical analysis was performed using the GraphPad Prism version 7. A statistical significance were considered *p*-values < 0.05.

## 3. Results

### 3.1. Characterization of the PLCL Scaffold 

Molecular weight and dispersity of PLCL measured using Gel Permeation Chromatography before and after electrospinning did not show any differences in PLCL properties. The parameters such as number average molecular weight (M_n_), weight average molecular weight (M_w_) and dispersity (Ð) are presented in Table 2. 

The hydrolytic modification of the PLCL nanofiber mat allowed the hydrophobic mat (with a water contact angle (WCA) of 116–133°) to become super-hydrophilic, with a WCA of 0°. The slightly alkaline hydrolysis enriched the hydrophobic surface of the PLCL polyester in carboxylic sodium salt and the hydroxyl groups, thus rendering it highly hydrophilic. In addition, the weight of the PLCL nanofiber mat decreased after the hydrolytic modification by 35%, which led to an increase in porosity from 65% to 75% (Table 3).

**Figure 1:**
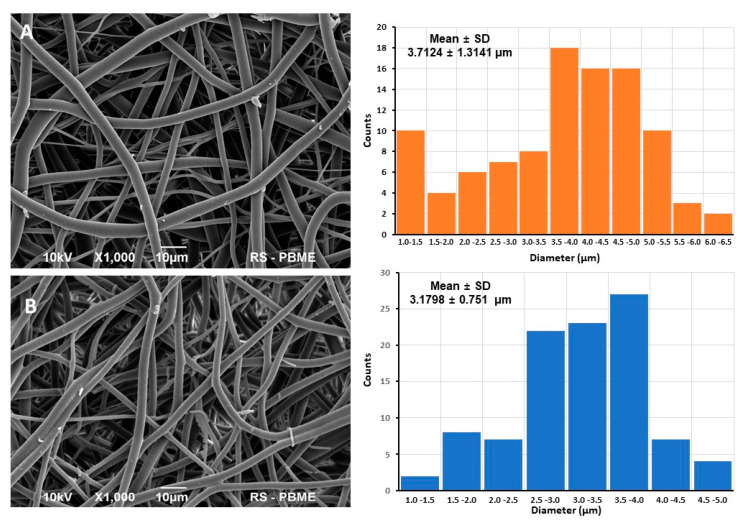
Scanning electron microscopy images of (**A**) PLCL before and (**B**) after modification and the corresponding nanofiber diameter distribution determined by measuring 100 fibers from SEM images. Scale bar = 10 µm.

Figure 1 show the top-view and cross-sectional SEM images of the electrospun PLCL nanofiber mat before (Figure 1A) and after (Figure 1B) the hydrolytic modification along with their diameter distribution. The average fiber diameter of the bare PLCL nanofiber mat was 3.7124 ± 1.3141 μm, while that of the hydrolytically-modified PLCL nanofiber mat was 3.1798 ± 0.7510 μm; the differences were statistically significant (*p* = 0.0003) (Table 3) (Figure 1A,B). Details for the electrospinning conditions for the PLCL nanofiber mat are described in the Experimental section. There was a slight reduction of 14% in the diameter of the PLCL nanofibers after the hydrolytic modification. The most affected were the thinnest fibers. Their volume fraction decreased from 35% to 25%. The average thickness of the PLCL nanofiber mat was not significantly altered during the hydrolytic modification and remained close to 120 μm. Considering the mechanical properties of PLCL with further in vitro experiments in mind, we decided to use the hydrolytically modified mat.

### 3.2. Morphological Features of the hDPSC Culture

hDPSCs were isolated from the third molars of healthy human participants and expanded in an in vitro culture in the α-MEM medium. Four days after seeding, the adherent hDPSCs showed a spindle-shaped fibroblast-like morphology and the ability to form colonies consisting of three–four cells. They displayed a large variation in cell morphology and growth potential (Figure S1A). After eight days from seeding, the morphology of the hDPSCs was heterogeneous and varied between cuboidal and spindle-shaped. After the first passages, the hDPSCs showed spindle-shaped, fibroblast-like cells similar to bone marrow mesenchymal stem cells (Figure S1B). The expanded hDPSCs revealed a good proliferative potential and the ability to achieve confluence in a flask in a short time.

### 3.3. Immunophenotypes of Cells Isolated from Dental Pulp Tissue 

The antigenic profile of cells isolated from dental pulp tissue was assessed using flow cytometry. We found that the hDPSCs expressed markers confirming the characteristics of the naïve MSC phenotype. More than 99% of the hDPSCs expressed CD73 (range 99.4–99.9%), CD90 (range 99.5–99.9%) and CD105 (range 72.0–99.7%) compared to isotype control. Additionally, a substantial population of primary hDPSCs expressed the typical MSC marker STRO-1 (range 38.5–84.5%) and CD44 (range 85.0–99.7%), and a part of the population of hDPSCs revealed CD54 (ICAM-1) (range 32.1–47.0%) and CD106 (VCAM-1) (range 12.0–21.0%) expression. According to the MSC definition, the cells were HLA ABC-positive (range 84.0–98.0%) and HLA DR-negative. MSC phenotype for the hDPSCs was confirmed by a lack of the expression of the common leukocyte marker CD45 (Figure S2).

### 3.4. Evaluation of hDPSC Multipotency

The hDPSCs differentiated into osteoblasts showed osteocalcin and osteopontin expression and revealed strong staining with Alizarin Red S (Figure S3A). The hDPSCs differentiated into chondrocytes showed positivity for Alcian Blue staining (Figure S3C). Oil Red O staining confirmed the adipogenic differentiation capacity of hDPSCs within 28 days of cultivation in a dedicated adipogenic medium (Figure S3E). Undifferentiated hDPSCs, serving as a control, are presented in Figure S3B,D,F.

### 3.5. Morphology of hDPSCs Growing on the Membrane

**Figure 2:**
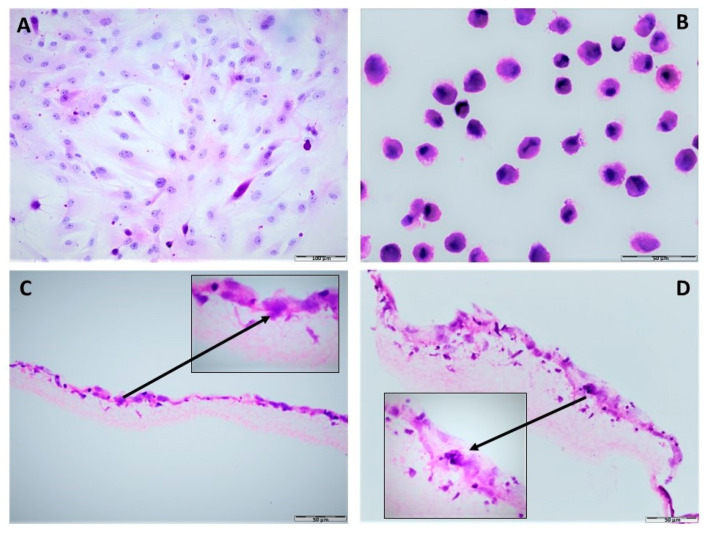
Histological analysis of the hDPSCs-PLCL construct after seven days of culture. (**A**) adherent hDPSCs morphology grown as a monolayer, spindle shape of cells is visible (**B**) cytospin specimen, morphological features of hDPSCs (**C**) hDPSCs covering the PLCL surface and individual cell infiltrating the PLCL nanofibers (**D**) a large number of hDPSCs infiltrate the deeper layers of the scaffold. Arrows indicate cells. Hematoxylin-eosin staining. Scale bar = 100 µm (**A**) and 50 µm (**B**–**D**).

Microscopic evaluation of the hDPSC-PLCL construct sections stained by hematoxylin and eosin showed that stem cells were present on and inside the scaffold. The morphological features of the hDPSCs growing in flasks as a monolayer (Figure 2A), such as the size and shape of cells, the location of the nucleus, and cytoplasm size, were comparable with the hDPSCs grown on the PLCL scaffold. The hDPSCs with defined morphological features (Figure 2B) were loaded onto the PLCL membrane and cultured for seven days. After this time point, the stem cells on the PLCL showed similar features to the cells grown in flasks. Moreover, the hDPSCs grown for seven days on the PLCL scaffold were able to attach to and spread onto the electrospun fibers covering most of the membrane surface. They were also equally distributed over the entire surface of the membrane and infiltrated the deep layers of the PLCL scaffold (Figure 2C,D). 

### 3.6. Expression of Surface Antigens in hDPSCs Growing on the Membrane

The expression of CD44, CD90, CD105 and Stro-1 was analyzed on hDPSCs before and after seven days of growth on the PLCL scaffold. As expected, the immunohistochemical data of the hDPSCs-PLCL construct samples showed no significant differences in the expression of the specific MSCs antigens assessed the hDPSCs cultures as a monolayer in the flasks and in the samples of the hDPSC-PLCL construct (Table 4).

**Figure 3:**
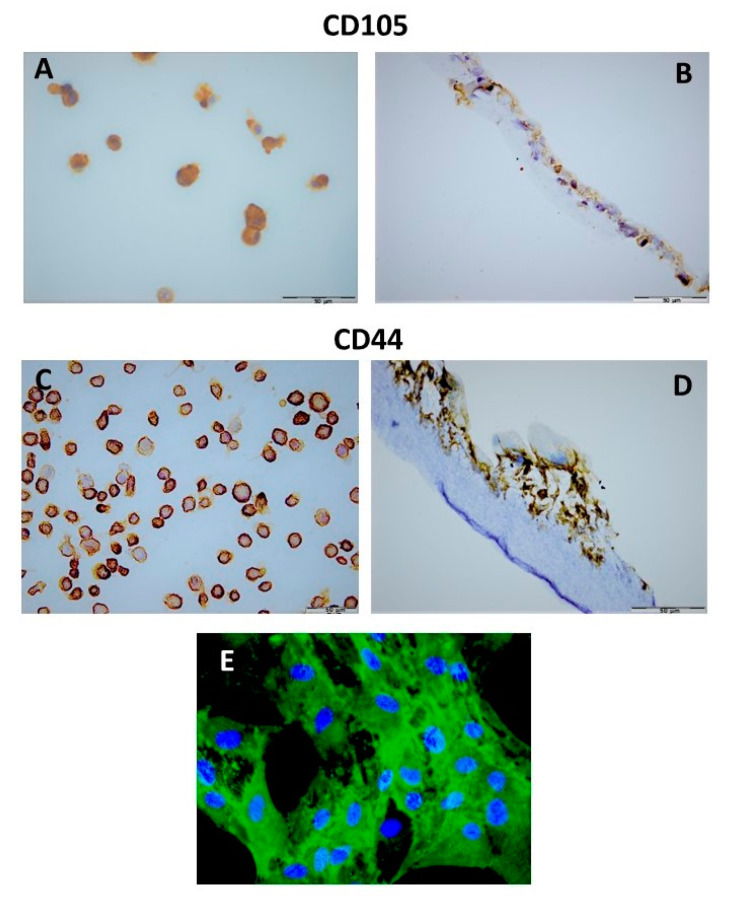
hDPSCs grown as a monolayer and on PLCL mat for seven days immunohistochemically stained for CD105 and CD44. (**A**) membrane expression of CD105 on hDPSCs grown as a monolayer (**B**) and grown on PLCL. (**C**) high CD44 expression detected in the majority of hDPSCs grown as a monolayer (**D**) and on the PLCL scaffold, (**E**) Immunofluorescence staining for CD44 marker (green) and DAPI (blue) revealed nuclei of hDPSCs grown as monolayer. (EnVision (**A**–**D**) and Immunofluorescence (**E**) techniques). Scale bar = 50 µm for (**A**–**D**) and magnification ×600 for (**E**).

However, a heterogeneous pattern of immunoreactivity was observed in the hDPSCs grown both as a monolayer and on the scaffold. The majority of hDPSCs revealed a strong immunopositivity for CD44 and CD105 (Figure 3A–E); however, a weak expression for the CD90 and STRO-1 markers was observed in a low percentage of the hDPSCs (10–20% of positive stem cells). To confirm the results, immunofluorescence staining was performed to assess the expression of stem cell biomarkers across the surface of the hDPSCs-PLCL construct. The results showed a similar positivity for the analyzed markers to that of the paraffin-embedded sample of the hDPSCs grown on PLCL; the expression of the CD44 marker was also observed (Figure 3E).

### 3.7. Viability, Adhesion and Spreading of hDPSCs Grown on the PLCL Scaffold

**Figure 4:**
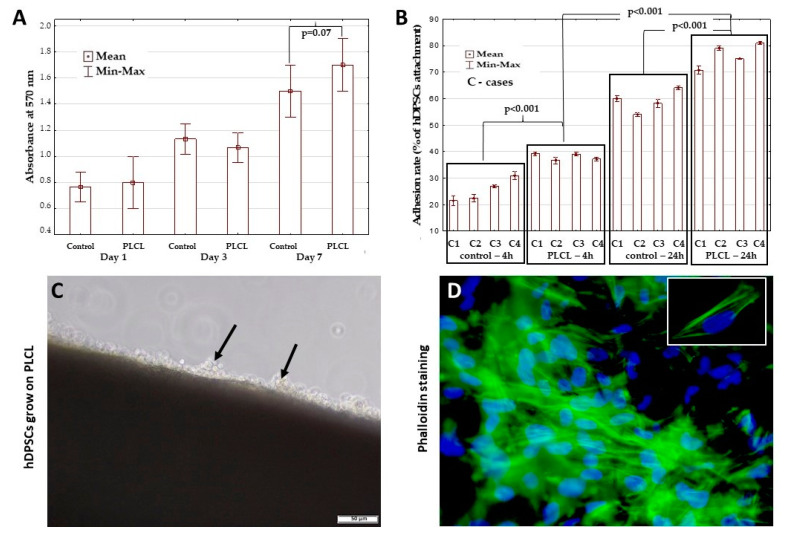
Viability and adhesion of hDPSCs grown on PLCL. (**A**) MTT assay results for hDPSCs grown on PLCL and as a monolayer culture (control) after one, three and seven days of culture. Borderline differences were found between the control group and the hDPSCs grown on PLCL on day seven of culture (*p* = 0.07). (**B**) adhesion rate of hDPSCs cultured on the PLCL nanofibrous scaffold was significantly higher compared to the control group at 4 and 24 h. Data are expressed as the mean ± SD (*n* = 4). (**C**) adhesion of hDPSCs seeded on the surface of PLCL (arrows) after 24 h of culture observed under inverted light microscopy. (**D**) Immunofluorescence staining of hDPSCs grown on PLCL scaffold by using Alexa Fluor 488 conjugated phalloidine is shown in green. Nuclei counterstained with DAPI are shown in blue. Actin structures are visible in the majority of cells growing on PLCL. In the white rectangle, there is a single enlarged hDPSC cell documenting the expression of actin. Scale bar = 50 µm (**C**) and magnification ×600 (**D**).

The impact of PLCL on hDPSCs viability was investigated using the MTT assay on days one, three and seven of cultivation. Figure 4A present the results as an average from all donors. According to MTT data, the viability of hDPSCs grown on the PLCL scaffold increased over time. The viability of the hDPSCs grown on PLCL was comparable with the control group on days one and three but slightly higher than the control group on day seven of cultivation. However, observed differences had only borderline significance (*p* = 0.07) (Figure 4A).

**Figure 5:**
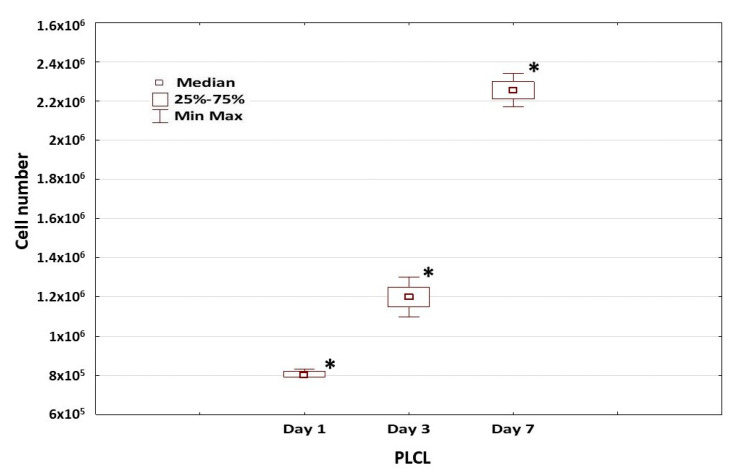
Spreading of hDPSCs grown on the PLCL scaffold. The number of hDPSCs grown onto PLCL significantly increased over time of cultivation (* *p* < 0.03). Columns show median spreading of hDPSCs ± SD. Three repeated assays were performed for each case (*n* = 4). Data are presented as median ± SD (*n* = 4).

Adhesion of the hDPSCs collected from the four donors and seeded on the PLCL scaffold was assessed 4 and 24 h after seeding. The adhesion rate of the hDPSCs (*n* = 4) in the scaffold group was significantly higher, ranging from 36.67% ± 0.49% to 39.22% ± 0.28% at 4 h and from 70.77% ± 0.61% to 80.97% ± 0.20% at 24 h, and ranging from 21.4% ± 0.79% to 30.92% ± 0.66% at 4 h and from 54.0% ± 0.29% to 64.07% ± 0.33% at 24 h in the control group (*p* < 0.001) (Figure 4B). After 24 h in hDPSC culture, the seeded cells were visible on the bridge of the PLCL scaffold (Figure 4C). In addition, diffuse phalloidin-stained structures found in the hDPSCs grown on the PLCL scaffold confirmed the presence of filamentous actin in cells (Figure 4D). The number of hDPSCs attached to the PLCL scaffold was associated with the cultivation time and significantly increased after 24 h of the culture of hDPSCs on the PLCL construct compared to 4 h (*p* < 0.001) (Figure 4B). The adhesion rate of the hDPSCs also differed between the individual cases at 4 and 24 h of cultivation, as confirmed by the statistical level (*p* < 0.001), except between cases 2 and 4 at 4 h of culture. Figure 5 presents the assessment of the hDPSCs spreading on the PLCL scaffold after 1, 3 and 7 days of cultured. The higher efficacy of spreading over larger areas on the PLCL scaffold was observed on day 7 compared to days 1 and 3 (Figure 5). Additionally, the number of cells on the PLCL surface increased up to day 7 and was significantly higher compared to days 1 and 3 (*p* < 0.03) (Figure 5). 

### 3.8. Cell Proliferation of hDPSCs Grown on the Membrane

**Figure 6:**
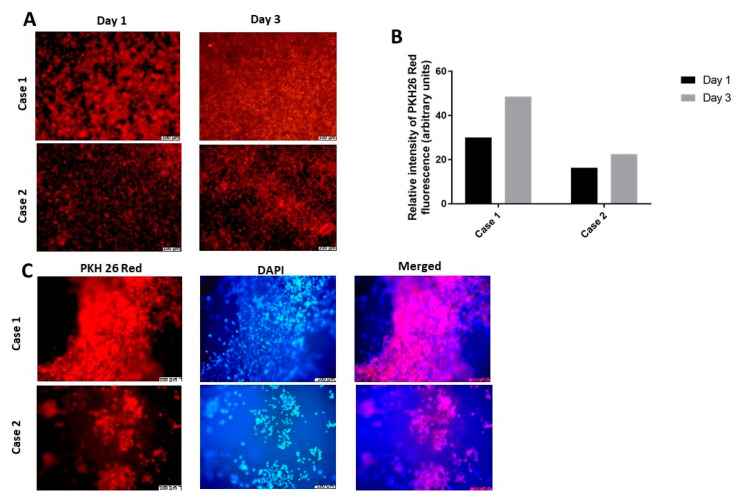
Proliferation activity of hDPSCs obtained from two cases (**A**) representative images of PKH26 red staining for hDPSCs from two cases three days after the scaffold seeding. (**B**) PKH26 red fluorescence intensity quantified on days one and three. (**C**) representative images of hDPSCs moved onto glass coverslips illustrate the extensive live colonies after three days of culture on the scaffold, as confirmed using PKH26 red and DAPI staining. Scale bar = 100 µm.

To assess cell proliferation on the PLCL scaffold, hDPSCs from two cases were stained with the PKH26 Red lipophilic membrane dye and monitored using fluorescence microscopy after one and three days of incubation. After 24 h of hDPSC cultivation on the scaffold, the cell density in case 1 was higher than in case 2 (Figure 6A). Nevertheless, cell proliferation on the scaffold was effective in both cases, as confirmed by an increase in PKH26 Red fluorescence intensity on day three of observation (Figure 6B). Images recorded on glass coverslips after three days of incubation showed that hDPSCs from both cases formed extensive live colonies, as confirmed by both PKH26 and DAPI staining (Figure 6C).

### 3.9. Doubling of the hDPSC Population

**Figure 7:**
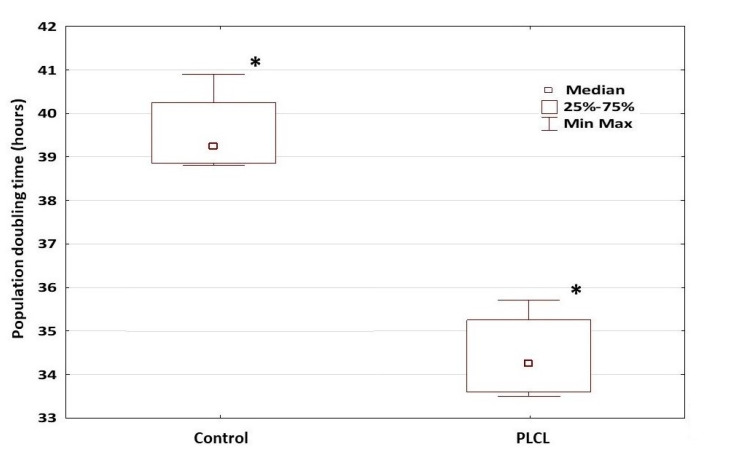
Population doubling time (PDT) efficiency. PDT of hDPSCs cultured on PLCL showed significantly lower PDT than hDPSCs cultured as a monolayer (* *p* < 0.01). Columns illustrate median PDT ± SD. Three independent PDT assays were performed for each case (*n* = 4).

An analysis of the population doubling time (PDT) of hDPSCs grown on PLCL and in flasks (as a control group) showed that the cells collected from each donor and grown on PLCL exhibited lower PDT (34.7 ± 1.11 h, *n* = 4) than the hDPSCs grown as monolayers (39.8 ± 1.06 h, *n* = 4) (*p* < 0.01). Figure 7 present the final PDT for both groups represented as the accumulated PDT obtained for each donor and the results of PDT as an average for the PLCL and the control group.

### 3.10. Osteogenic Differentiation of hDPSCs on PLCL Scaffold, Mineralization, Bone-Related Proteins Expression

The status of differentiated cells was confirmed by Alizarin Red S staining and mRNA levels of bone-related genes (*osteocalcin (OCN), osteopontin (OPN), dentin sialophosphoprotein (DSPP), bone sialoprotein (BSP)*) and their protein expression. Immunohistochemistry was used to analyze the expression of OCN, OPN, BSP and DSPP proteins on the hDPSCs/PLCL paraffin block section, the surface of hDPSCs/PLCL constructs and cytospin specimens.

**Figure 8:**
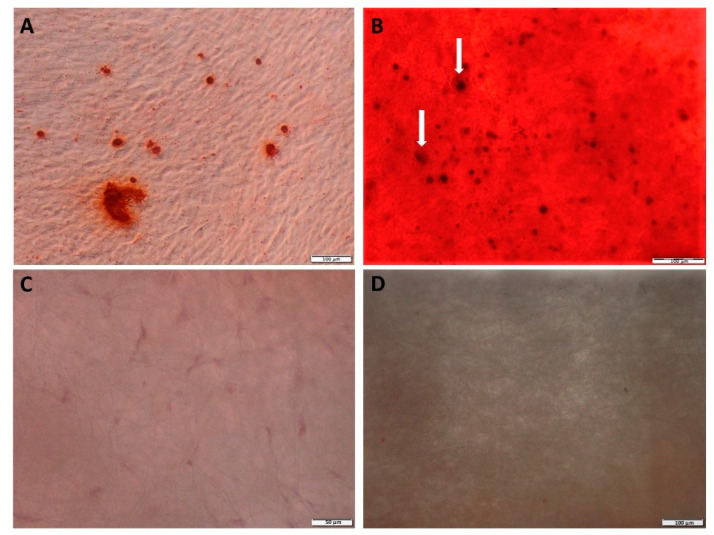
Alizarin Red S staining of hDPSCs on 2D culture dish and on PLCL scaffold after osteogenic differentiation. (**A**) Mineralized nodules after hDPSCs osteogenic differentiation in 2D culture, (**B**) hDPSCs are covered and embedded in mineralized matrix, and mineral deposits on PLCL surface stained by Alizarin Red S are visible (arrows). Control staining of (**C**) PLCL with non-differentiated hDPSCs and (**D**) PLCL without hDPSCs illustrate a lack of Alizarin Red S staining. Scale bar = 100 µm (**A**,**B**,**D**) and 50 µm (**C**).

Alizarin Red S staining was employed to analyze the calcium deposition in hDPSCs differentiated towards osteoblasts on the PLCL scaffold after 21 days cultured under osteogenic differentiation medium. The deposition of mineralized extracellular matrix and calcium mineralization was confirmed by Alizarin Red S staining. As shown in Figure 8A, hDPSCs grown as 2D were positive for Alizarin Red S staining, and mineralized nodules were visible. hDPSCs after differentiation on PLCL were embedded within the cell-produced mineralized matrix, resulting in calcium deposits, and single red mineralized nodules were observed on the PLCL surface (Figure 8B). Control staining of PLCL with non-differentiated hDPSCs and PLCL without hDPSCs was negative for Alizarin Red S staining (Figure 8C,D).

**Figure 9:**
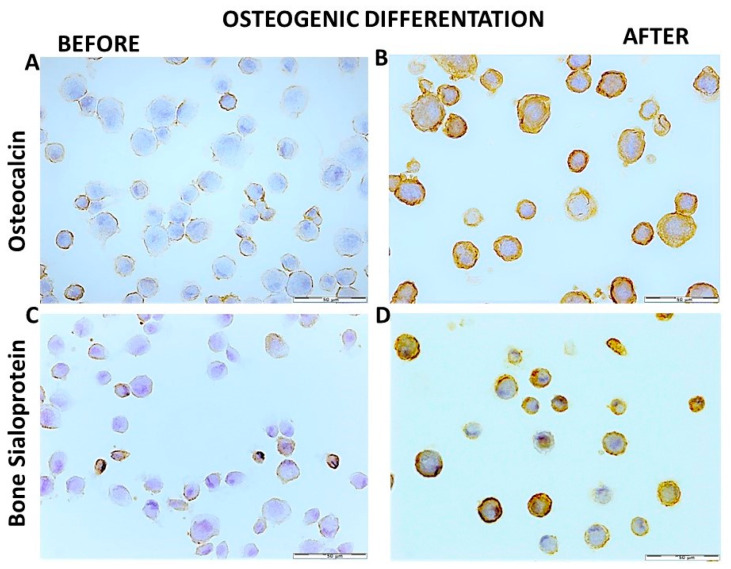
Comparison of bone-related proteins expressed by hDPSCs grown on PLCL nonfibrous scaffold before and after osteogenic differentiation. (**A**) Cytospin specimens of hDPSCs showed weak expression of OCN before (**B**) and high OCN expression after osteogenic differentiation. (**C**) hDPSCs demonstrated low expression of BSP before (**D**) and intensive expression of BSP on hDPSCs after differentiation. (EnVision technique). Scale bar = 50 µm.

Immunohistochemical findings showed significant differences between BSP, OCN, OPN and BSSP protein expression of hDPSCs grown on PLCL before and after osteogenic differentiation (Table 5). hDPSCs before osteogenic differentiation showed weaker expression of bone-related proteins, resulting in a low percentage of positive cells for analyzed proteins compared to differentiated cells (Figure 9A–D).

**Figure 10:**
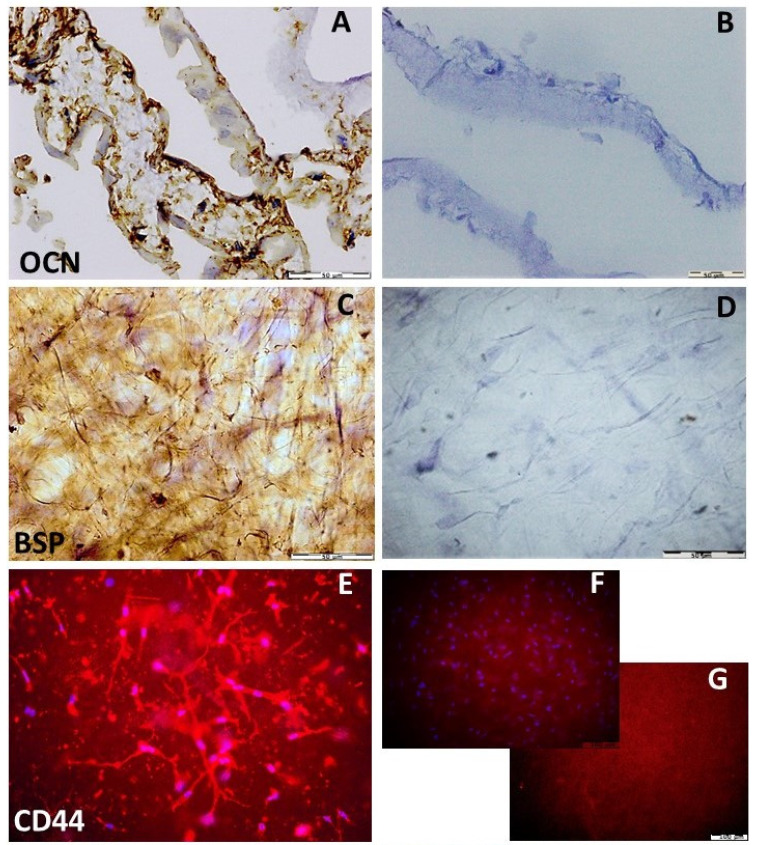

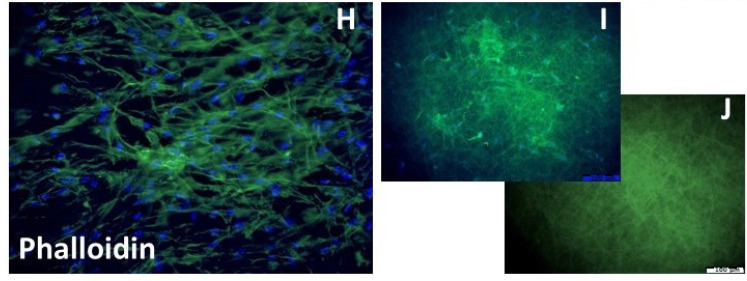
Osteogenic differentiation ability of hDPSCs on PLCL nanofibrous scaffold analyzed by immunohistochemical staining for bone-related proteins expression after osteogenic differentiation. (**A**) strong expression of OCN showed by hDPSC on PLCL in paraffin block section. (**B**) control case negative IHC staining. (**C**) BSP expression in hDPSCs growing on PLCL surface. (**D**) control case negative IHC staining. (**E**) immunofluorescence image of CD44 expression in differentiated hDPSCs on PLCl. Controls of immunofluorescence staining: (**F**) PLCL membrane with hDPSCs without primary antibody CD44 stained with secondary antibody marked TRITC and (**G**) PLCL membrane alone without primary antibody CD44 stained with secondary antibody marked TRITC. (**H**) Phalloidin staining observed in a majority of differentiated hDSPSC on PLCL. Controls of immunofluorescence staining: (**I**) PLCL membrane with hDPSCs without primary antibody stained with secondary antibody Alexa Flour 488 and (**J**) PLCL membrane alone without primary antibody stained with secondary antibody Alexa Flour 488. Envision technique (**A**–**D**) and immunofluorescence (**E**–**J**) technique, Scale bar = 50 µm (**A**–**D**) and 100 µm (**F**,**G**,**I**,**J**) and magnification ×200 (**E**,**H**).

The differentiated hDPSCs on the PLCL construct expressed the OPN, OCN, BSP and DPSS proteins in the majority of cells. The expression of all biomarkers showed a heterogeneous pattern of immunostaining, and immunopositive cells were found throughout the surface of the scaffold, as presented in Figure 10A,C. Background IHC staining was not observed in fibers of the PLCL scaffold (Figure 10B,D). Moreover, the differentiated hDPSCs on PLCL showed adhesion to PLCL surface confirmed by CD44 immunostaining (Figure 10E), and filaments stability was revealed by strong phalloidin staining (Figure 10H). However, PLCL with seeded hDPSCs (Figure 10F,I) and PLCL without hDPSCs (Figure 10G,J) showed weak autofluorescence background.

### 3.11. Osteogenic Differentiation of hDPSCs on PLCL Scaffold, qRT-PCR Analysis

**Figure 11:**
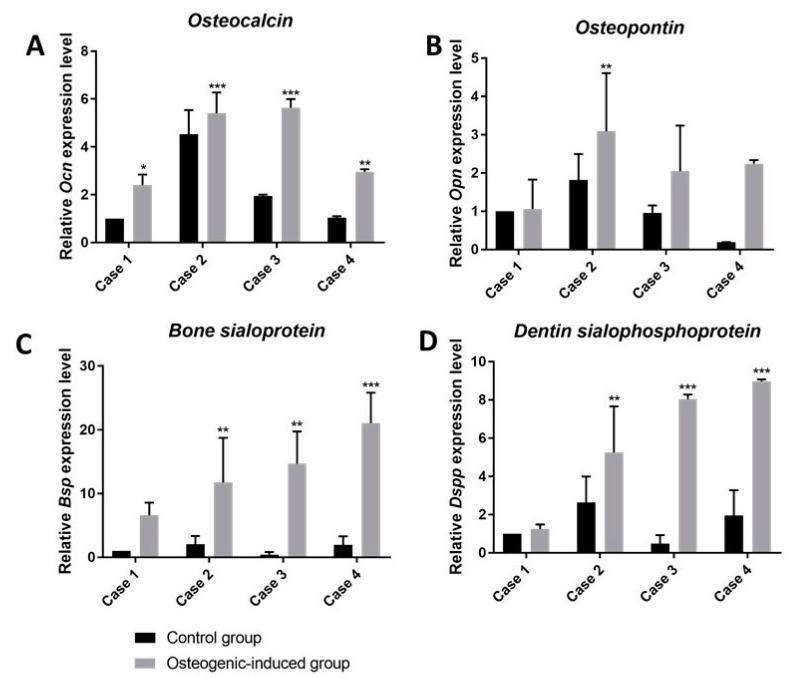
Real-time PCR analysis of the bone-related genes in osteogenic induced hDPSCs-PLCL construct (*n* = 4) and undifferentiated hDPSCs-PLCL (control group, *n* = 4). (**A**) *Osteocalcin* (*OCN*) was significantly upregulated in all cases compare to control group (* *p* < 0.05, ** *p* < 0.005, *** *p* < 0.0001). (**B**) *Osteopontin* (*OPN*) expression significantly increased in one cases (** *p* < 0.005). (**C**) *Bone sialoprotein* (*BSP*) and (**D**) *Dentin sialophosphoprotein* (*DSPP*) genes were significantly upregulated in three of four hDPSCs/PLCL constructs (** *p* < 0.005, *** *p* < 0.0001).

The gene expression of four osteogenic differentiation markers: (*OCN*), (*OPN*), (*BSP*) and (*DSPP*) in hDPSCs after osteogenic differentiation and undifferentiated hDPSCSs was analyzed. The relative expression level of *osteocalcin* increased in all cases after the osteogenic induction of hDPSCs. Interestingly, *OCN* expression of non-induced hDPSCs in Case 2 was at a relatively high level, compared to others (RQ 4.53, *p* < 0.0001) (Figure 11A). However, the greatest increase of *OCN* expression levels between the control and the osteogenic-induced group was observed in Case 3 (RQ 1.95 vs. 5.64, *p* < 0.0001) (Figure 11A). *Osteopontin* gene expression levels increased in all examined cases, except Case 1 (RQ 1.00 vs 1.06) (Figure 11B). The highest relative *OPN* expression level was detected for the osteogenic induced group of Case 2 (RQ 3.09, *p* < 0.005) (Figure 11B). The relative gene expression level of *bone sialoprotein* significantly increased in all cases in the osteogenic-induced group compared to the control. The highest *BSP* level of the osteogenic-induced group was observed in Case 4 (RQ 21.06, *p* < 0.0001) (Figure 11C), and the lowest level in Case 1 (RQ 6.58) (Figure 11C). *Dentin sialophosphoprotein* expression level increased in all osteogenic-induced groups compared to the control. However, similar to *OPN*, in Case 1, the difference between the control and osteogenic-induced group was marginal (RQ 1.00 vs. 1.26) (Figure 11D). In the osteogenic-induced group of Case 4 was the highest *DSPP* expression level (RQ 8.97, *p* < 0.0001) (Figure 11D). Nevertheless, the most expressed *DSPP* level between the control and osteogenic-induced group was observed in Case 3 (RQ 0.48 vs. 8.04, *p* < 0.0001) (Figure 11D), similar to *OCN* expression.

## 4. Discussion

Recent studies report that the dental pulp contains a population of stem cells, such as hDPSCs, which express antigens characteristic for MSCs isolated from different tissue sources of mesodermal origin [14,34,50]. This study confirmed that the adherent fraction of the hDPSCs displays the typical characteristics and immunophenotype of MSCs through the expression of the CD44, CD73, CD90, CD105 and STRO-1 markers and a lack of the hematopoietic markers CD45 and HLA-DR. The MSC characteristics of the hDPSCs were also proven through their tri-lineage differentiation potential into the osteo-, chondro-, and adipogenic lineage [50].

hDPSCs constitute a heterogenic MSC population and are frequently assessed for their differentiation abilities into odontogenic, osteogenic and neurogenic tissues [23]. However, for successful tissue-specific regeneration, it is important to use an appropriate scaffold that mimics the extracellular matrix in the native tissue and helps to increase the regenerative potential of the hDPSCs. This study characterized the biological properties of hDPSCs. It tested whether an electrospun nanofibrous PLCL membrane could be a suitable scaffold for hDPSC adhesion, proliferation, immunophenotype stability and osteogenic differentiation to obtain a bioactive construct for use in tissue engineering. Based on early data showing that the mechanical properties of PLCL can be improved by modifying the fibers to achieve a higher porosity of the membrane, which increases cell colonization and proliferation on their surface [41,43,44], we introduced, for the first time, a hydrolytically-modified PLCL nanofiber mat as a scaffold for hDPSC growth. In this study, the hydrolytic modification of a mat consisting of a poly(l-lactide-*co*-caprolactone) copolymer revealed similar changes to those observed by other authors [5,39,45]. After the fiber hydrolysis, a significant mass loss occurred with only a slight increase in porosity and a decrease in average fiber diameter compared to a non-modified PLCL mat found in this study. These results are comparable with other data showing that PLCL blending with silk causes a rapid loss of mass, increased surface roughness and improved hydrophilicity compared to a pure PLCL scaffold [45]. On the other hand, in agreement with previously published data, the mechanical properties of modified PLCL used in our study may provide an appropriate space for hDPSC growth and facilitate nutrient transportation inside a scaffold [39,45]. We confirmed that PLCL, which increased porosity and hydrophilicity, can benefit the biological behavior of hDPSCs on their surface, as was shown for other stem cells sources [5,44,45]. The proliferative assay confirmed that the hDPSCs were able to grow and proliferate on the PLCL membrane. Furthermore, the viability of the hDPSCs increased over time, and on day 7 of culture, it was higher than in the control. The presented results proved that PLCL was not toxic to dental pulp stem cells and that both components of the PLCL scaffold had a beneficial effect on hDPSC viability [5,6]. In our experimental study, we used a PLCL scaffold with similar physical and chemical features, including average fiber diameter, mass loss of the mat, porosity, and water contact angles, to those described by other investigators [39,41,44,45]. In the current study, hDPSCs revealed good adhesion to the PLCL scaffold, and the number of attached stem cells depended on the time of culture. These results suggest that fiber thickness and mat porosity, without a discernible morphological degradation of PLCL, facilitate hDPSC attachment to the scaffold surface and enhance hDPSC colonization [44,45]. Moreover, thanks to the highly hydrophilic properties of modified PLCL, the mat improved binding between the hDPSCs and the functional groups on their surface [5,45]. This observation is consistent with a previous report showing that the mechanical and chemical parameters of a modified PLCL scaffold may enhance the adhesion and proliferation of stem cells isolated from human subcutaneous adipose tissue (ADSCs) [45]. In addition, Alipour et al. [39] revealed that the high porosity of a poly(caprolactone)—poly(ethylene glycol)—poly(caprolactone) scaffold is important for hDPSC growth. The high spreading of hDPSCs on the surface of the PLCL scaffold and their ability to migrate into the deeper layers of the mat, presented in this study, indicate that PLCL demonstrates appropriate surface roughness and nanofibrous architecture and porosity suitable for protein absorption from the culture medium, which is necessary for hDPSC attachment and growth [39,40,45]. The high hDPSC proliferation and their ability to infiltrate the hydrolytically-modified PLCL, presented in this study, suggest that hDPSC behavior on the PLCL scaffold may be associated with the components of this construct and its degradation [3,5,6,38,40]. However, the rate of scaffold degradation depends on the components and their proportion in the scaffold. According to Bazgir et al. [52], PCL nonwoven electrospun membrane of thickness 0.11 mm lost about 20% of its weight for 12 weeks degradation in PBS in a room temperature. Pogorielov et al. [53] found that PCL nanofibrous matrices lost 38 ± 5% of their mass during degradation in simulated body fluid for 12 weeks. Moreover, scaffold made of PLCL (67% l-lactide and 33% ε-caprolactone composition have lost 29.8% of mass during in vitro 36 weeks degradation in saline at 37 °C, and sponge made of 70/30i-lacticde/ε-caprolactone copolymer (PLCL) seeded with myoblast undergone complete in vivo degradation 9 months after implantation [44,54]. This observation is in line with early experimental studies, which showed that poly(lactide) (PLA) had good biocompatibility with stem cells collected from exfoliated deciduous teeth (SHED) and that the environment generated by PLA degradation was effective for stem cell adhesion and viability [4,6]. Likewise, polycaprolactone (PCL), as a scaffold for MSCs and hDPSCs, has a positive impact on their growth and proliferation [5,39,55,56,57]. A similar effect to that reported by other authors, i.e., a high capacity of hDPSCs to spread over large areas of the scaffold, was observed in this study when both materials were used to construct the scaffold [39,45]. These results indicate that the modified PLCL scaffold induces a suitable microenvironment for hDPSC growth. Additionally, this study found that the spreading of the hDPSCs on PLCL was associated with the rate of hDPSC proliferation, as confirmed by the number of hDPSCs stained with PKH26. These data indicate that modified PLCL enhances not only the spreading of hDPSCs, but also their proliferation [45]. Published data suggest that the biological behavior of hDPSCs on PLCL can be explained by the ability of stem cells to push the individual fibers in the electrospun mat away to create the space necessary for cell proliferation [40,45]. This ability of hDPSCs to create space by pushing the nanofibers aside was confirmed in this study by the morphological analysis of the hDPSCs-PLCL construct. To our knowledge, this is the first study to assess the morphological features of hDPSCs grown on a scaffold and their migration into a PLCL mat that involves the analysis of the hDPSCs-PLCL construct in a paraffin-embedded block. This technique allows for the analysis of the larger area of the scaffold surface and its deeper layers compared to analysis performed using a scanning microscope [4,20,27]. Our data showed that PLCL does not induce changes in the morphology of hDPSCs independent of cultivation time, but also found that hDPSCs growing on PLCL scaffold showed good attachment to the fibers and were capable of migrating inside the micropores of the nanofibrous membrane, as was observed by other investigators [4,40]. Moreover, we found that the extent of hDPSC migration into the scaffold structure depended on the time of hDPSC growth on PLCL and their proliferation. These results indicate that PLCL degradation induces a microenvironment that promotes dental stem cell distribution, proliferation, and viability [45]. Based on the previous data, the presented results suggest that the active groups of the PLCL fibrous scaffold may cooperate with hDPSCs and determine their biological behavior [5,45]. In addition, this study revealed the ability of hDPSCs to migrate inside PLCL and suggest that this scaffold provides a porous structure inside the stent for nutrient and oxygen transport that supports hDPSC growth and spread onto and inside the scaffold [5,6,40].

To date, there are no reports on a comparative analysis of the immunophenotype of hDPSCs cultured on a PLCL scaffold and grown as a monolayer. Nevertheless, the data presented in this study proved that the features of MSCs found in hDPSCs can be confirmed through immunopositivity for the CD105, CD90, CD73, CD44 and STRO-1 markers and their ability to differentiate into the chondro-, adipo- and osteogenic lineages [4,7,12,16]. A comparative analysis of the expression of MSC biomarkers on hDPSCs grown on PLCL and in standard conditions did not reveal any significant differences. These results indicate that PLCL creates proper environmental conditions for the hDPSC immunophenotype stability. These findings are partly comparable with reports presenting the immunophenotype stability of hDPSCs or MSCs differentiation into osteoblasts [23,39]. Our data and previously published data suggest that the hydrolytically-modified PLCL nanofiber mat scaffold possesses suitable parameters for hDPSC growth and phenotype stability [6,39]. Moreover, the stable immunophenotype of hDPSCs is one important feature that has an impact on hDPSCs osteogenic differentiation. The smart scaffold for bone tissue engineering should not only promote proliferation and adhesion but also facilitate the osteogenic differentiation of seeded stem cells and generate biomineralization of ECM [39,45]. To investigate the usefulness of PLCL nanofibrous scaffold for dentin bone tissue engineering, the potential of osteogenic differentiation of hDPSCs on PLCL was examined. Similarly to published reports in our study, hDPSCs/PLCL construct showed strong staining for Alizarin Red S suggesting positive effect of PLCL nanofibrous in stimulation of ECM mineralization [5,39,45]. We found that during osteogenic differentiation, hDPSCs release extracellular deposits, which form mineralized nodules. This ability is an important feature for evaluating the function of hDPSCs and is crucial for bone-forming [6]. Moreover, we noted that strong Alizarin Red S staining observed on the PLCL surface indicates high mineralization, which is usually associated with the osteo-like phenotype of cells located on their surface [39]. Our results also revealed that PLCL mat promote good adhesion of differentiated hDPSCs to PLCl surface and ensure the stability of cytoskeleton structure of differentiated hDPSCs [55].,

Performed in the current study, analysis of bone-related gene expression indicated that hDPSCs on the PLCL nonfibrous scaffold differentiated into osteoblast-like cells. Our data showed that both the mRNA levels of osteogenic-related genes (*OCP*, *OPN*, *BSP*) and their proteins expression increased in hDPSCs seeded on PLCL after osteogenic differentiation compared to the control group. This data indicated that DPSCs grown on PLCL scaffold could be differentiated into cells showing an osteoblast immunophenotype and secrete a mineralized bone-like matrix. In line with other reports, we might conclude that PLCL nonfibrous scaffold provided a favorable microenvironment for the intracellular signaling activity in cell–cell and cell–extracellular matrix interactions and affected osteogenic differentiation of hDPSCs and mineralization of ECM [45,46]. Based on the presented results, bioconstruct designed of adherent hDPSCs to PLCL nonfibrous scaffold showing high proliferation, osteogenic potency and mineralization ability seems to be a promise graft materials for bone tissue engineering [39]. Taking into account a study by Jundziłł et al. [41] showing that a pure PLCL scaffold induced angiogenesis after six weeks of implantation into the rat model, we are considering investigating this ability of a hDPSCs-PLCL construct in a future study performed in vivo on an animal model. The new study will test the differentiation capacity of hDPSCs into functional cells, including chondrocyte, osteoblast and neuronal cells, onto a PLCL scaffold as a bioactive construct for tissue engineering in dentistry and medicine.

## 5. Conclusions

In conclusion, these results showed that the mechanical properties of a modified PLCL mat provide an appropriate environment that supports hDPSC attachment, proliferation, migration and osteogenic differentiation of hDPSCs growing on PLCL scaffold. The good PLCL biocompatibility with dental pulp stem cells indicates that this nanofibrous scaffold may be applied in designing a bioactive hDPSCs/PLCL construct for bone tissue engineering, and it is worthy of further exploration.

## Figures and Tables

**Table 1:** Genes and primers used in qRT-PCR assays.

Target Gene	NCBI Reference Sequence	Sequence (5′–3′)	Amplicon Length (bp)
Osteopontin (OPN)	NM_001251830.1	F:5′-ATCACCTGTGCCATACCA-3′ R:5′-CATCTTCATCATCCATATCATCCA-3′	1823
Osteocalcin (OCN)	NM_199173.4	F:5′-GCAGGTGCGAAGCCCAGCGGTGCAGAG-3′ R:5′-GGGCTGGGAGGTCAGGGCAAGGGCAAG-3′	562
Bone sialoprotein (BSP)	NM_004967	F:5′-TCACTGGAGCCAATGCAGAA-3′ R:5′-TGGAGAGGTTGTTGTCTTCGAG-3′	1573
Dentin sialophosphoprotein (DSPP)	NM_014208.3	F:5′-GGCAGTGCATCAAAAGGAGC-3′ R:5′-TGCTGTCACTGTCACTGCTG-3′	4331
β-actin (β-actin)	NM_001101.3	F:5′-AGGGCAGTGATCTCCTTCTGCATCCT-3′ R:5′-CCACACTGTGCCCATCTACGAGGGGT-3′	1852

**Table 2:** Properties of PLCL before and after electrospinning.

	Number Average Molecular Weight (Mn)	Weight Average Molecular Weight (Mw)	Dispersity (Ð)
PLCL before electrospinning	35 kDa	134 kDa	3.9
PLCL after electrospinning	34 kDa	130 kDa	3.8

**Table 3:** Properties of electrospun scaffolds before and after the modification.

	Water Contact Angle	Mass Loss	Porosity	Mean Average Fiber Diameter	*p*
Unmodified PLCL fibers	116–133°	-	65%	3.7124 ± 1.3141 μm	0.0003
Modified PLCL fibers	0°	35%	73%	3.1798 ± 0.7510 μm	

**Table 4:** Comparison of mesenchymal stem cell markers expression on hDPSCs growing onto PLCL scaffold and as a monolayer.

Cultured Condition	Immunoreactivity (Percentage of Positive Cells, Mean +SD)								
	Number of Donors	CD105		CD90		CD44		Stro-1	
		*n* [%]	*p*	*n* [%]	*p*	*n* [%]	*p*	*n* [%]	*p*
hDPSCs grown in flasks	4	86.7 + 13.591		57.1 + 20.048		93.7 + 5.818		16.2 + 10.232	
			NS		NS		NS		NS
hDPSCs grown on PLCL	4	91.2 ± 5.052		59.2 ± 24.301		94.6 ± 5.575		10.4 ± 5.187	

hDPSCs- human dental pulp stem cells; *n* [%]—mean (%) ±SD of positive hDPSCs; *p*—student *t*-test; NS—non statistically significant differences.

**Table 5:** Comparison of bone-related proteins expression on hDPSCs growing onto PLCL before and after differentiation towards osteoblasts.

Cultured Condition	Immunoreactivity (Percentage of Positive Cells, Mean ±SD)								
	Number of Donors	BSP		OCN		OPN		DSPP	
		*n* [%]	*p*	*n* [%]	*p*	*n* [%]	*p*	*n* [%]	*p*
hDPSCs on PLCL before differentiation	4	13.9 ± 4.581	<0.0003	23.3 ± 5.271	<0.0005	22.8 ± 9.75	<0.0001	11.1 ± 4.581	<0.0001
hDPSCs on PLCL after differentiation	4	45.6 ± 14.99		61.1 ± 19.689		62.2 ± 17.498		46.7 ± 8.165	

*n* [%]—mean (%) ± SD of positive hDPSCs; *p* ≤ 0.05 statistically significant differences, *p*—student *t*-test.

## Data Availability

The data presented in this study are available on request from the corresponding authors.

## Supplementary Materials

The following supporting information can be downloaded at: https://www.mdpi.com/article/10.3390/ma15051900/s1, Figure S1: Morphological characterization of hDPSCs isolated from dental pulp tissue and cultured as a monolayer, as examined under an inverted light microscope. (A) Cultured hDPSCs exhibited typical fibroblast-like cell morphology and adherence to plastic culture dishes five days after seeding. (B) hDPSCs grown into colonies after 12 days of seeding. Scale bar = 100 µm; Figure S2: Representative flow cytometry analysis of hDPSC phenotype. Adherent cells show the basic naïve MSC phenotype CD73+/CD90+/CD105+ and express specific mesenchymal stem cell marker Stro-1 and cell adhesion molecule CD44, part of the population express ICAM-1, VCAM-1. hDPSCs are positive for the HLA ABC antigen and negative for HLA DR and CD45 expression. Red-filled histograms correspond to MSCs labelled for the analyzed antibodies, and empty histograms illustrate isotype controls; Figure S3: Tri-lineage differentiation of hDPSCs. (A) Osteogenic mineralization was assessed by Alizarin Red S staining (C) chondrogenic-proteoglycans were stained by Alcian Blue, (E) adipogenic-lipid vesicles were revealed by Oil Red O positive staining; (B,D,F) control cells cultured in non-differentiation α-MEM medium. Scale bar = 100 µm.
