# Supplementary material for: Characterization of Biological Properties of Dental Pulp Stem Cells Grown on an Electrospun Poly(l-lactide-co-caprolactone) Scaffold
Source: Materials (Basel). 2022 Mar 3;15(5):1900. doi: 10.3390/ma15051900 (PMC8911644; doi:10.3390/ma15051900)
Supplement: Supplementary file 1 [file materials-15-01900-s001.zip › materials-1390512-supplementary.pdf]

materials

Supplementary Materials

Characterization of Biological Properties of Dental Pulp Stem Cells Grown on an Electrospun Poly(L-lactide-co-caprolactone) Scaffold

Julia K. Bar [1,*,†], Tomasz Kowalczyk [2], Piotr G. Grelewski [1], Sandra Stamnitz [3], Maria Paprocka [3], Joanna Lis [4], Anna Lis-Nawara [1], Seongpil An [5] and Aleksandra Klimczak [3,†]

[1] Department of Immunopathology and Molecular Biology, Medical University, Bujwida 44, 50-345 Wroclaw, Poland; piotr.grelewski@umed.wroc.pl (P.G.G.); anna.lis-nawara@umed.wroc.pl (A.L.-N.)
[2] Laboratory of Polymers and Biomaterials, Institute of Fundamental Technological Research (IPPT PAN), Polish Academy of Sciences, Adolfa Pawińskiego 5B St., 02-106 Warsaw, Poland; tkowalcz@ippt.gov.pl
[3] Laboratory of Biology of Stem and Neoplastic Cells, Hirszfeld Institute of Immunology and Experimental Therapy Polish Academy of Sciences, R. Weigla 12, 53-114 Wroclaw, Poland; sandra.gromolak@hirszfeld.pl (S.S.); maria.paprocka@hirszfeld.pl (M.P.); aleksandra.klimczak@hirszfeld.pl (A.K.)
[4] Department of Maxillofacial Orthopaedics and Orthodontics, Medical University, Krakowska 26, 50-425 Wroclaw, Poland; joanna.lis@umed.wroc.pl
[5] Department of Nano Engineering & SKKU Advanced Institute of Nanotechnology (SAINT), Sungkyunkwan University (SKKU), Suwon 16419, Korea; esan@skku.edu
* Correspondence: julia.bar@umed.wroc.pl; Tel.: +48-697770614
† J.K.B. and A.K. have equal contributions.

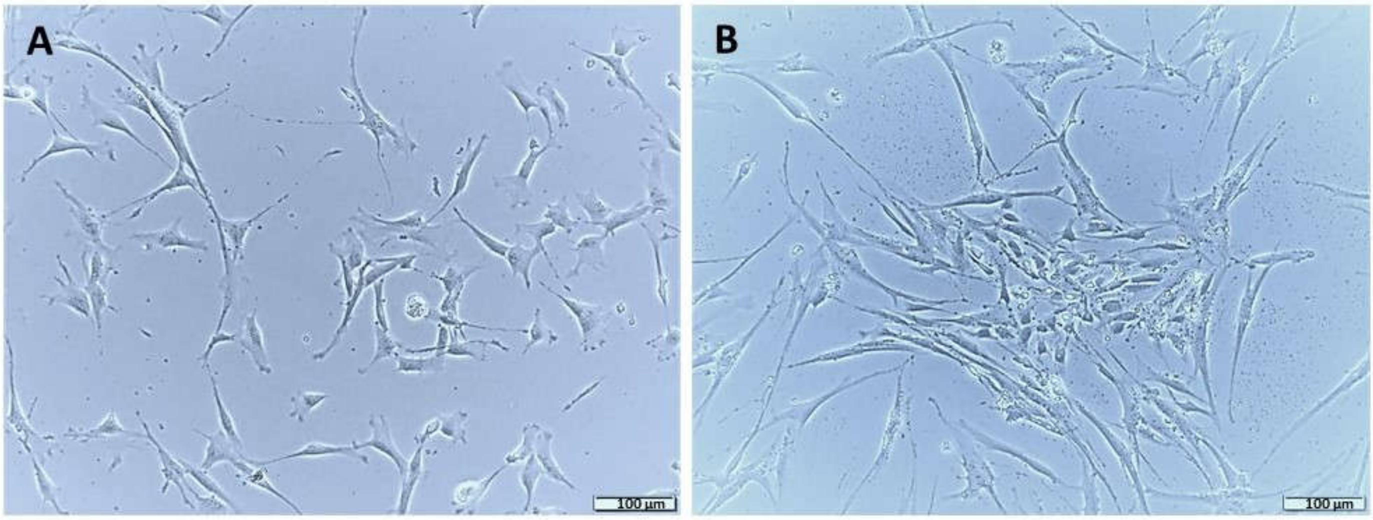

Figure S1. Morphological characterization of hDPSCs isolated from dental pulp tissue and cultured as a monolayer, as examined under an inverted light microscope. (**A**) Cultured hDPSCs exhibited typical fibroblast-like cell morphology and adherence to plastic culture dishes five days after seeding. (**B**) hDPSCs grown into colonies after 12 days of seeding. Scale bar = 100 µm.

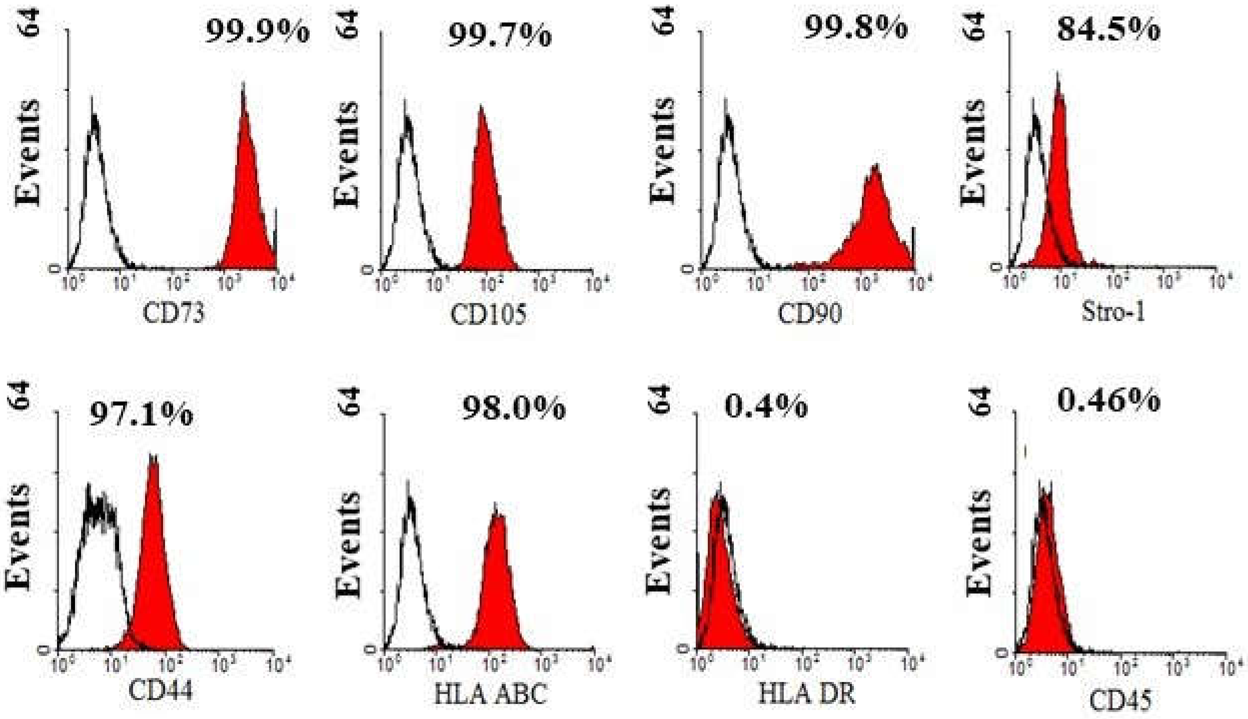

Figure S2. Representative flow cytometry analysis of hDPSC phenotype. Adherent cells show the basic naïve MSC phenotype CD73+/CD90+/CD105+ and express specific mesenchymal stem cell marker Stro-1 and cell adhesion molecule CD44, part of the population express ICAM-1, VCAM-1. hDPSCs are positive for the HLA ABC antigen and negative for HLA DR and CD45 expression. Red-filled histograms correspond to MSCs labelled for the analyzed antibodies, and empty histograms illustrate isotype controls.

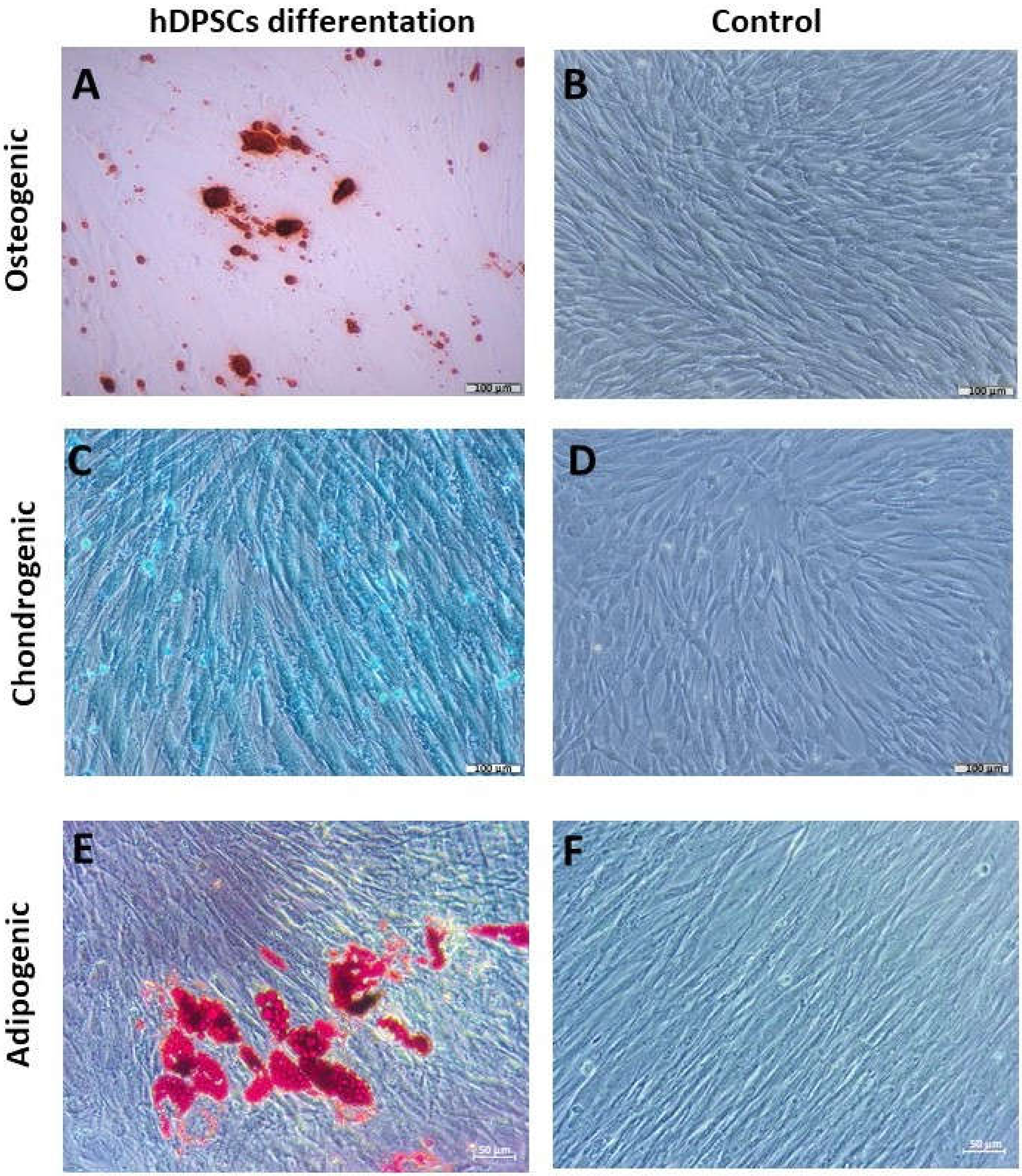

Figure S3. Tri-lineage differentiation of hDPSCs. (**A**) Osteogenic mineralization was assessed by Alizarin Red S staining (**C**) chondrogenic-proteoglycans were stained by Alcian Blue, (**E**) adipogenic-lipid vesicles were revealed by Oil Red O positive staining; (**B**,**D**,**F**) control cells cultured in non-differentiation α-MEM medium. Scale bar = 100 µm.
